# Engaging Chinese and Korean American communities in dementia research: A journey of inclusivity and partnership

**DOI:** 10.1002/alz.70664

**Published:** 2025-10-17

**Authors:** Jing Wang, Xiang Qi, Mary S. Mittelman, Eunjung Ko, Yaolin Pei, I. Tek Leong, SungJi Park, Katherine Wang, Weiyu Mao, Cynthia Epstein, Bei Wu

**Affiliations:** ^1^ College of Health and Human Services University of New Hampshire Durham New Hampshire USA; ^2^ Rory Meyer College of Nursing New York University New York New York USA; ^3^ Departments of Medicine and Population Health, NYU Grossman School of Medicine New York New York USA; ^4^ School of Nursing University of Texas at Austin Austin Texas USA; ^5^ Albert Einstein College of Medicine Bronx New York USA; ^6^ School of Social Work University of Nevada Reno Nevada USA; ^7^ NYU Shanghai Shanghai China

**Keywords:** cardiometabolic risks psychosocial intervention, community partnerships, recruitment

## Abstract

**INTRODUCTION:**

The New York University Caregiver Intervention plus Enhanced Support Project is a randomized controlled trial of a family‐based psychosocial intervention to enhance social support and reduce cardiometabolic risk for Chinese and Korean American dementia caregivers, using culturally tailored recruitment strategies.

**METHODS:**

We reviewed reflections from research staff, weekly meeting minutes, debriefing sessions, and progress reports, to identify key challenges and approaches to engaging participants.

**RESULTS:**

Key challenges included reluctance to involve family members, dementia stigma, and resistance to involving family. In response, we engaged online communities, partnered with local organizations, participated in events, and adapted recruitment messages to cultural norms. For the Chinese community, we focused on practical skills while for the Korean community, we emphasized caregiving strategies and the personal/social benefits of participation, reducing rejection rates.

**DISCUSSION:**

Our findings underscore the importance of culturally tailored recruitment strategies in dementia research. Respectful, sensitive, and culturally informed approaches can significantly enhance engagement and participation.

**Highlights:**

- Culturally adapted recruitment strategies improve study engagement with Chinese and Korean American dementia caregivers.
- Community partnerships with local social services agencies are essential for recruitment success.
- Culturally relevant social media applications were integrated to increase accessibility for study participants.
- This study uniquely targets and recruits Chinese and Korean American dementia caregivers with metabolic syndrome‐related symptoms, incorporating a psychological intervention alongside biomarker data collection.
- The iterative adaptation of recruitment methods and tailored messaging to specific ethnic groups ensure the intervention is culturally aligned, enhancing both participation and relevance to the caregivers’ unique health and caregiving contexts.

## 1 BACKGROUND

The number of older Americans living with Alzheimer's disease and related dementias (ADRD) is projected to increase to 13.8 million by 2060.^1^ ADRD disproportionately affects racial and ethnic minority populations who often experience unique dementia etiologies, prognoses, and care patterns.^2^ A population‐based study found that the age‐adjusted incidence of dementia was significantly higher among older Asian American adults than among White adults.^3^ Despite these disparities, ethnic minority populations, particularly Asian Americans, remain significantly underrepresented in dementia research.^4^ Historically, the model minority stereotype has portrayed Asian Americans as being in good health, contributing to the underrepresentation of their health needs in clinical research.^5^ This perception overlooks the specific needs of those with limited English proficiency and creates a gap in culturally appropriate care strategies.

Chinese Americans and Korean Americans represent two of the largest and fastest‐growing subgroups of Asian Americans at risk for ADRD.^6^, ^7^ In addition to elevated dementia risk, many Asian American family caregivers face intersecting barriers, including high rates of cardiometabolic conditions, limited access to bilingual mental health services, and cultural stigma surrounding dementia and caregiving.^8^, ^9^ These overlapping challenges are especially pronounced in urban areas like New York City, where nearly half of Asian Americans report limited English proficiency, yet linguistically and culturally appropriate services remain scarce.^10^, ^11^, ^12^ These factors, coupled with systemic issues like a lack of bilingual researchers and clinicians and inadequate outreach efforts,^13^, ^14^, ^15^ have resulted in significant gaps in both research and health‐care interventions for these populations. This underrepresentation in dementia research is exacerbated by misconceptions about dementia and a lack of culturally competent services.^13^, ^16^

Recent scholarship has highlighted the importance of cultural competence and community engagement in research with underserved populations. Effective recruitment approaches often involve long‐term relationships with community stakeholders, involvement of cultural insiders, and outreach strategies that prioritize trust, relevance, and accessibility.^17^, ^18^ Nguyen et al. emphasize the value of culturally appropriate outreach and community partnerships, which are reflected in our recruitment strategy.^19^ In our approach, we partnered with community members to host culturally tailored meetings, collaborated with trusted community leaders, and used word‐of‐mouth referrals to enhance participation.

Our project is based in New York City (NYC), which enables opportunities for trust building and localized outreach that may not be as readily available in other regions. For example, in March 2025, we co‐hosted a press event with CaringKind that attracted representatives from nine community organizations and reporters from four major Chinese‐language newspapers—*World Journal, Sing Tao Daily, China Press*, and *Epoch Times*.  CaringKind and Korean Community Services of Metropolitan New York (KCS) were chosen as our two primary partners. CaringKind provides dementia consultations, caregiver training, and education programs tailored for diverse communities, while KCS offers older adult services, mental health support, and intergenerational community programs for Korean Americans in the area. Studies by Liu et al.^20^, ^21^, ^22^ further validate this approach, showing that culturally grounded recruitment strategies can effectively engage Chinese American dementia caregivers in research based in NYC.

The New York University (NYU) Caregiver Intervention plus Enhanced Support (NYUCI‐ES) Project addresses the specific challenges faced by Chinese and Korean American dementia caregivers through a culturally sensitive recruitment strategy centered on community partnerships. Building on the proven success of the original NYU Caregiver Intervention (NYUCI),^23^, ^24^ NYUCI‐ES extends these outcomes to underserved communities. The project focuses on improving health outcomes for caregivers who are at high risk for cardiometabolic issues by delivering a family‐based psychosocial intervention. This intervention is designed to enhance social support and improve physical health by mitigating cardiometabolic risks.

The objective of this study is to analyze and explore the challenges and strategies involved in the recruitment of Chinese and Korean American caregivers in the NYUCI‐ES Project. In this paper, we share interim results of our ongoing recruitment efforts, offering insights gained along the way. We aim to underscore the critical role that culturally tailored recruitment strategies play in increasing research participation.

## 2 METHODS

### 2.1 Overview of the P50 NYUCI‐ES project

The NYUCI‐ES project aims to promote psychological well‐being and physical health among Chinese and Korean American dementia family caregivers. The NYUCI‐ES is culturally adapted from the existing NYUCI, an evidence‐based program for families caring for individuals living with dementia, which has shown significant improvement in various aspects of caregiver well‐being, such as satisfaction with social support,^25^ burden,^26^ the reduction in stress and caregivers’ depressive mood,^27^ a decrease in reactions to care recipients’ behavior problems,^28^ and delayed replacement to nursing homes.^29^ Fauth et al.^26^ addressed this intervention as having an external validity in diverse community settings, although there have been low participation rates of racial minority populations, including Asian Americans, in previous studies of the NYUCI.

Family counseling, a key component of NYUCI, is particularly relevant for Chinese and Korean American families due to cultural values of filial piety and family harmony.^30^ It aligns with the family‐centered caregiving approach common in many Asian cultures, in which caregiving decisions and responsibilities are shared among family members.^31^ Family counseling also helps mediate conflicts that may arise during caregiving, fostering a more cohesive support system for the primary caregiver.^32^ NYUCI‐ES includes all the core elements of the original intervention with added cultural adaptations. Participants are randomly assigned to a treatment group, which receives two individual counseling sessions, four family counseling sessions, ad hoc counseling, and culturally relevant online group support, or to a control group that receives ad hoc counseling and resource information.

**RESEARCH IN CONTEXT**

1. **Systematic review**: The authors reviewed recent literature on recruitment and retention strategies for ethnic minority populations, with a focus on Asian American dementia caregivers. Searches were conducted in PubMed for peer‐reviewed journal articles addressing culturally tailored approaches, community partnerships, and the use of technology in recruitment processes. Key studies on bilingual staff, cultural competence, and health interventions were examined to understand best practices.
2. **Interpretation**: This study provides valuable insights into the challenges and opportunities of fostering inclusive recruitment among Chinese and Korean American dementia caregivers. It underscores the importance of cultural adaptation, including the use of familiar social media applications, community partnerships, and culturally sensitive communication, to ensure equitable participation in dementia caregiving research. Our findings highlight the critical role of these strategies in reaching underrepresented groups, ensuring their involvement in interventions aimed at addressing the diverse needs of caregivers and reducing health disparities.
3. **Future directions**: Future research should prioritize developing sustainable, culturally tailored recruitment strategies that foster long‐term engagement among ethnic minority caregivers. A key focus should be on establishing strong community partnerships and tailoring messages to the specific cultural contexts of different ethnic groups. Additionally, expanding recruitment efforts across diverse ethnic groups will enhance understanding of caregiver stress and health outcomes, advancing equitable health‐care interventions and improving support for caregivers from all backgrounds.

Before launching NYUCI‐ES, we conducted pilot interviews with Chinese and Korean American dementia caregivers and social service providers to better understand the cultural context and specific needs of our target population.^30^ Findings revealed that caregivers’ perceptions of burden, support seeking, and privacy were deeply influenced by cultural values such as filial piety, face saving, and stigma related to mental illness and dementia. These interviews informed the design of our culturally tailored recruitment and engagement protocol. For example, many caregivers expressed reluctance to disclose emotional strain or family conflict, fearing judgment or loss of face. In response, we emphasized confidentiality and relational trust building in our outreach materials and interactions. We avoided the use of stigmatizing terms such as “mental health” or “counseling” in favor of more neutral, culturally acceptable language like “family support” or “well‐being conversations.” We also allowed participants to bring fictive kin (e.g., close friends or church members) to family counseling sessions when they felt uncomfortable involving immediate family. To increase accessibility and comfort, we included direct contact information, including staff phone numbers, in our recruitment materials and allowed flexible communication through social media platforms such as WeChat and KakaoTalk. We also incorporated culturally relevant practices, such as offering tea and refreshments during sessions, and using appropriate forms of respectful address and tone. Six bilingual team members (three Chinese and three Korean) led recruitment and communication efforts. We also included two licensed social workers of matching ethnic backgrounds, who played a significant role in establishing rapport and sustaining participant engagement across interviews, counseling sessions, and follow‐up conversations. Despite these tailored efforts, we encountered early challenges in messaging and terminology that required further adaptation. This iterative process underscored the need to remain flexible and responsive to cultural cues throughout recruitment and implementation. The evolving protocol highlights the importance of continuous refinement in cross‐cultural intervention research.

NYUCI‐ES takes place in the New York Metropolitan Area, home to large Chinese and Korean American populations. Bilingual licensed clinical social workers collect data through three in‐person visits at baseline, 6‐month, and 12‐month follow‐ups. Measures include demographic characteristics, caregiving context, emotional health (loneliness, depressive mood, stress), social support usage, and physical health (self‐reported and objective measures such as blood pressure, body mass index, and blood tests with consent). By incorporating culturally sensitive components into the NYUCI model, NYUCI‐ES aims to meet the unique needs of Chinese and Korean American ADRD caregivers, improving both psychological and physical health outcomes.

### 2.2 Overview of the recruitment

#### 2.2.1 Eligibility criteria

Participants eligible for the NYUCI‐ES should be individuals who meet the following criteria: (1) self‐identified as a primary caregiver of individuals with memory problems; (2) at high risk of cardiometabolic symptoms, such as diabetes, hypertension, high cholesterol, overweight/obesity, or other metabolic syndrome‐related diseases; (3) aged ≥ 50 years old; (4) live in the New York metropolitan area; (5) are not planning to move out of state within the next 12 months; (6) have internet access; (7) are able to read English, Chinese, or Korean; and (8) report that they have at least one family member who can attend family counseling sessions. Exclusion criteria were (1) history of psychiatric diagnoses requiring hospitalization during the past 5 years and (2) not able to complete the informed consent.

#### 2.2.2 Recruitment goals

Considering power analysis to detect a treatment arm difference in a key inflammation biomarker at a single post intervention visit point, the targeted number of participants is 300, including 150 for treatment (75 Chinese, 75 Korean) and 150 for the control from February 2023 to July 2026. The study was approved by the institutional review board at NYU Grossman School of Medicine.

#### 2.2.3 Description of recruitment strategies

Recruitment started in February 2023. We initially recruited eligible Chinese and Korean American caregivers through community organizations with which we have built strong partnerships. Our recruitment of Chinese caregivers heavily relied on referrals from CaringKind, an organization that runs an extensive dementia outreach program within the local Chinese community. Several members of our team serve on the advisory board for this program. Our recruitment of Korean American caregivers relied largely on Korean Community Services of Metropolitan New York. Among the fourteen team members, six were bilingual (Chinese/English or Korean/English) and directly involved in recruitment and participant communication. This included three Chinese and three Korean bilingual staff, two of whom were licensed social workers who shared the same ethnic and linguistic backgrounds as the participants. These bilingual staff played an integral role in establishing trust, conducting outreach, and facilitating ongoing communication throughout the study. Participants received a total of $150 to $200 in gift cards over the course of the 1 year study period, depending on their level of involvement. This compensation acknowledged their time and contributions to interviews, counseling sessions, and feedback activities.

We expanded our recruitment sources in July 2023 (the details of our recruitment sources are in Table 1). Participants are now being recruited through multiple avenues including, but not limited to: memory clinics, local health fairs in partnership with community organizations, clinical practices focused on serving Asian patients, e‐mail listservs, flyers, and chain recruitment. We also promote this project via other community resources in the New York Metropolitan Area by using e‐mail listservs, distributing flyers, attending local health fairs for Chinese and Korean American populations, providing educational materials, advertising on social media, attending volunteering activities, and holding press releases for the recruitment. We also collaborate with the Collaborative Approach for Asian Americans and Pacific Islanders Research and Education (CARE) registry^4^ and encourage recruitment through word of mouth. Upon referral from community resources or calls from individuals interested in the NYUCI‐ES, we begin the screening process to check their eligibility. If the individuals do not answer, we make three to five follow‐up calls.

**TABLE 1:** Summary of recruitment sources.

Recruitment sources	Description	The number of participants enrolled	% of total enrolled participants by resource
Community organizations	Organizations for CA (e.g., CaringKind, Asian Community Care Organization [ACCO], Hamilton Madison House [HMH] house caregiver program, Korean Community Services of Metropolitan New York [KCS])	75 CA	70.83%
	Organizations for KA (e.g., KCS)	122 KA	
	Hospitals or clinics (Mount Sinai, VNS Health)	7 CA 0 KA	
Word of mouth	Referring our study to their network, peer, or someone else who will be eligible for the study	50 CA 5 KA	19.10%
Community outreach	Attending community events, volunteering, and hosting educational workshop in communities	21 CA 0 KA	7.29%
Social media	Posting project recruitment advertisement (flyer, poster, etc.) to forums in local websites or WeChat official accounts or contacting local newspapers to host the news press release	5 CA 1 KA	2.08%
Other	Distributing our project advertisement to local churches/libraries	2 CA 0 KA	0.69%
	Using a research registry – collaborated with UCSF Collaborative Approach for Asian Americans & Pacific Islanders Research & Education (CARE; IRB No.19‐28027) to recruit underrepresented population (Asian Americans, Native Hawaiians, and Pacific Islanders) in research	0 CA 0 KA	0%

Abbreviations: CA, Chinese Americans; IRB, institutional review board; KA, Korean Americans; UCSF, University of California San Francisco.

### 2.3 Information sources and interpretation process

The information for this study was drawn from both narrative and quantitative sources to contextualize the challenges encountered during the recruitment process and to describe recruitment outcomes.

We collected narrative insights from weekly team meetings, structured reflections from investigators and research staff, debriefing session notes, field notes, and progress reports. These narrative materials offered contextual depth, shedding light on practical barriers, facilitators, and adaptations to our recruitment strategies. All descriptive themes related to recruitment challenges and strategies presented in this article were derived directly from team meeting minutes, structured reflections, field notes, and progress reports generated during the recruitment period of the current NYUCI‐ES Project. To systematically organize and synthesize this information, we adapted a matrix‐based approach, a method commonly applied in implementation research and program evaluation to support structured comparison and pattern recognition across data sources and participant groups.^33^, ^34^ Our matrix was organized by participant group (Chinese vs. Korean American), recruitment strategy, observed challenges, and the adaptations implemented. By organizing the data in this structured manner, we were able to examine patterns across data types and team perspectives, enhancing the credibility and consistency of our interpretation. Although we did not conduct formal qualitative coding or use software (e.g., NVivo or Atlas.ti), the adapted matrix approach enabled structured, team‐based synthesis of recurring insights. This facilitated an organized reflection on how recruitment strategies evolved in real time in response to emerging challenges and community feedback.

In addition, quantitative data were obtained from the progress report and screening evaluation used during participant intake, which captured demographic characteristics, caregiver–care recipient relationships, and self‐reported health status. These data were used to provide a descriptive summary of the participants and to explore differences between those who enrolled and those who were eligible but did not participate. To aid interpretation, we developed summary tables (Tables 2 and 3) comparing characteristics between participants and eligible non‐participants. We used Stata 18.0 for data analysis. A two‐sample *t* test was used for the continuous variable (age), and a Pearson chi‐squared test was used for categorical variables. The purpose of these comparisons is not to support inferential conclusions but to provide context about the study sample and explore potential differences between those who enrolled and those who did not. These descriptive comparisons help identify participation patterns and assess the potential for selection bias, that is, whether individuals who enrolled differed systematically from those who were eligible but declined participation.

**TABLE 2:** Comparison of eligible individuals’ characteristics by participation status.

	Eligible but not participating (*N* = 45)	Eligible and participating (*N* = 288)	*t* test or χ^2^ test (*p* value)^a^
Caregiver's age (mean, SD)	65.97 (11.91)	68.63 (9.40)	−1.5118 (*p* = 0.132)
Caregiver's sex – female (*N*, %)^b^	35 (79.55%)	240 (83.33%)	0.3851 (*p* = 0.535)
Relationship to care recipient – spouse or partner (*N*, %)	19 (42.22%)	161 (55.90%)	2.9329 (*p* = 0.087)
Status of diabetes – yes (*N*, %)	18 (40.91%)	106 (36.81%)	0.2747 (*p* = 0.600)
Status of high cholesterol – yes (*N*, %)	31 (70.45%)	204 (70.83%)	0.0026 (*p* = 0.959)
Status of hypertension – yes (*N*, %)	17 (38.64%)	160 (55.56%)	**4.3897 (** *p* ** = 0.036)**
Status of overweight or obesity – yes (*N*, %)	12 (28.57%)	135 (46.88%)	**4.9712 (** *p* ** = 0.026)**
Presence of more than one health status – yes, more than one (*N*, %)	26 (57.78%)	190 (65.97%)	1.1467 (*p* = 0.284)

*Note*: Values in bold indicate statistical significance at *p* < 0.05.

Abbreviations: N, number; SD, standard deviation.

^a^ A *t* test is for constant variables, while χ^2^ test is for nominal variables.

^b^ Respondents addressed their sex as either female or male. No other gender identities were reported.

**TABLE 3:** Comparison of participants’ characteristics by their origin (Chinese vs. Korean).

	CA (*N* = 160)	KA (*N* = 128)	Total (*N* = 288)	*t* test or χ^2^ test (*p* value)^a^
Caregiver's age (mean, SD)	69.22 (8.91)	67.89 (9.96)	68.63 (9.40)	1.1921 (*p* = 0.234)
Caregiver's sex – female (*N*, %)^b^	133 (83.12%)	107 (83.59%)	240 (83.33%)	0.0112 (*p *= 0.916)
Care recipient's sex – female (*N*, %)^b^	60 (39.47%)	57 (45.97%)	117 (42.39%)	1.1793 (*p* = 0.277)
Relationship to care recipient – spouse or partner (*N*, %)	95 (59.38%)	66 (51.56%)	161 (55.90%)	1.7606 (*p* = 0.185)
Status of diabetes – yes (*N*, %)	71 (44.38%)	35 (27.34%)	106 (36.81%)	8.8683 **(*p* = 0.003)**
Status of cholesterol – yes (*N*, %)	117 (73.12%)	87 (67.97%)	204 (70.83%)	0.9151 (*p* = 0.339)
Status of hypertension – yes (*N*, %)	87 (54.37%)	73 (57.03%)	160 (55.56%)	0.2032 (*p* = 0.652)
Status of overweight or obesity – yes (*N*, %)	103 (64.38%)	32 (25.00%)	135 (46.88%)	44.2729 **(*p* < 0.001)**
Presence of more than one health status – yes, more than one (*N*, %)	125 (78.12%)	65 (50.78%)	190 (65.97%)	23.6842 **(*p* < 0.001)**

*Note*: Values in bold indicate statistical significance at *p* < 0.05.

Abbreviations: CA, Chinese Americans; KA, Korean Americans; N, number; SD, standard deviation.

^a^ A *t* test is for constant variables, while χ^2^ test is for nominal variables.

^b^ Respondents addressed their sex as either female or male. No other gender identities were reported.

## 3 RESULTS

We shared interim results of our ongoing recruitment efforts, offering insights gained along the way, as recruitment is still in progress. We underscored the critical role that culturally tailored recruitment strategies play in increasing research participation among Chinese and Korean American dementia caregivers. From February 2023 to June 2025, a total of 288 Chinese and Korean American dementia caregivers were enrolled through a variety of community‐engaged strategies. Key challenges that impacted participation, such as cultural barriers, logistical concerns, and reluctance to involve family members are described, followed by the iterative strategies implemented to address these issues. We concluded by highlighting culturally tailored adaptations and engagement efforts that enhanced accessibility and improved participation among these historically underrepresented groups.

### 3.1 Challenges encountered

#### 3.1.1 Commitment to sessions and home visits

A small number of Chinese and Korean American caregivers who chose not to participate in the study raised concerns about the time commitment involved. The requirement of multiple sessions and home visits over a 12 month period felt challenging for some, particularly those with limited availability or who anticipated difficulty coordinating with family members.

Ten Chinese American caregivers addressed their concerns about a lack of time and found study protocol to be burdensome during the screening calls. Full‐time working adult children, in particular, felt that coordinating several sessions could add stress, especially when it involved negotiating meeting times with family members. Although the number of required visits was manageable for many, some participants hesitated due to concerns about balancing the participation with their existing responsibilities.

Similarly, Korean American caregivers felt uncertain about the family counseling sessions, particularly the four required for the intervention. Approximately 30 people refused to participate due to the time‐related issues with anticipated study‐related burden. Some indicated they didn't have family members or friends available to attend, and others felt uncomfortable asking their children, given their busy schedules. This made it more challenging for some participants to meet the family‐based requirements of the study. This also brought to our attention the significance of reframing the participation as culturally meaningful rather than simply focusing on the logistical aspects of the intervention, making it more relevant and appealing to potential participants.

#### 3.1.2 Reluctance to involve family members

More than 100 of the Chinese and Korean American caregivers declined to participate due to no family member joining the family counseling sessions, although the underlying reasons varied between the two groups. For both groups, there was a reluctance tied to the practicalities of involving family members. Among Chinese American caregivers, some spouses mentioned that their adult children were too busy or didn't see the need to participate if they couldn't provide direct help. A few adult children, on the other hand, felt uncomfortable discussing caregiving challenges with siblings or other family members. In contrast, for some Korean American caregivers, the hesitation was more tied to cultural expectations and concerns about burdening their children. For example, 14 Koreans expressed hesitation about involving family members in the study, and 3 even feared that telling their children about their care recipient's memory loss, which could add stress. This concern was particularly relevant for those worried about disrupting family dynamics, as some shared that they didn't want caregiving responsibilities to cause disputes, especially between their adult sons and their spouses.

#### 3.1.3 Cultural barriers toward caregiver support and counseling

Both Chinese and Korean American caregivers encountered barriers due to cultural stigma and unfamiliarity with counseling and caregiver support, which were further influenced by generational differences in openness to research participation. Generational differences played a critical role in this regard. Caregivers with lower levels of acculturation, particularly older individuals who are more rooted in traditional cultural practices, were less likely to understand or see the value in participating in a study involving family counseling. In contrast, caregivers with higher levels of acculturation, often younger and more educated, and with better English proficiency, were more open to research and more familiar with counseling as a form of support. This difference highlights the impact of acculturation on the perception and acceptance of mental health interventions like family counseling.

For Chinese American caregivers, discomfort with the concept of “counseling” was common, as many believed that support should be directed toward the person with dementia rather than themselves. Unfamiliarity with the notion of “caregiver support” also led to resistance, as discussing personal challenges or seeking external help is not a typical practice in Chinese culture. Cultural expectations that caregiving responsibilities fall on certain individuals, such as the spouse, eldest son, or unmarried daughter, often led caregivers to assume the role without considering the option of asking for support from other family members. As a result, family counseling was frequently viewed as irrelevant or unnecessary.

For Korean American caregivers, cultural stigma surrounding caregiving and counseling was even more pronounced. Many, especially those caring for in‐laws or parents, downplayed the difficulties of caregiving and were reluctant to acknowledge the challenges they faced. This reluctance was further compounded by unfamiliarity with counseling and equated it with therapy and did not understand its value in managing caregiver stress. Some caregivers expressed skepticism about counseling, questioning how it could address their day‐to‐day caregiving burdens. Additionally, several participants with strong religious beliefs preferred spiritual practices over counseling, stating a preference for prayer rather than discussing their hardships with a social worker or family members.

#### 3.1.4 Concerns about privacy and study participation

Concerns about privacy and the nature of study participation were common in both groups, though they manifested differently.

For Chinese American caregivers, privacy concerns related to home visits were particularly pronounced during the initial recruitment phase after the COVID‐19 pandemic. Many participants were uncomfortable with the idea of having a social worker visit their home.

For Korean American caregivers, privacy concerns led them to express a preference for joining the control group. Many potential participants did not want their children to know about their involvement in the study, fearing they might misunderstand the reasons for participation. Some were concerned that their children might think they were participating for financial gain or that it would imply they were wasting their time. This reluctance to disclose participation also made it challenging to involve family members in the counseling sessions, even among those who agreed to join the study.

#### 3.1.5 Blood test and health‐related concerns

Initially, we scheduled lab visits for participants interested in our study, offering incentives to encourage participation and cover travel expenses. However, both Chinese and Korean American caregivers expressed concerns about the need to travel to the lab, especially when the person with dementia lacked a home health aide or family members to assist. Health‐related concerns, particularly regarding blood tests, arose in both groups. When we adapted by offering at‐home visits with less invasive methods for blood collection, participants' concerns were significantly mitigated. In fact, this solution transformed the initial challenge into a motivating factor for some participants, a strategy that will be elaborated on in subsequent sections.

### 3.2 Iterative process of the cultural adaptation of recruitment strategies

Informed by ongoing participant feedback and team reflections, our recruitment approach was not static but evolved iteratively to respond to emerging challenges in real time. This process allowed us to remain flexible, culturally responsive, and attuned to the diverse needs of Chinese and Korean American caregivers. Rather than a one‐size‐fits‐all strategy, we used a multifaceted approach grounded in cultural humility, community engagement, and pragmatic adaptation. As shown in Table 4, each strategy is listed alongside the specific challenge(s) it was designed to address, providing a clear visual summary of our adaptation process.

**TABLE 4:** Challenges and strategies.

Challenge	Strategy	Specific strategy
3.1.1 Commitment to sessions and home visits	3.2.3 Reframing participation as culturally meaningful	Framed family meetings as opportunities for children to practice filial piety (Chinese) and emphasized personal and societal benefits of participation (Korean).
3.1.1 Commitment to sessions and home visits	3.2.4 Adapting delivery methods	Offered home visits and scheduling flexibility to reduce logistical burden.
3.1.2 Reluctance to involve family members	3.2.2 Culturally sensitive language adjustments	Replaced “counseling” with culturally resonant terms such as “family meeting” (Chinese) and “talk about caregiving strategies” (Korean).
3.1.2 Reluctance to involve family members	3.2.3 Reframing participation as culturally meaningful	Presented participation as family responsibility and contribution to community well‐being to reduce discomfort with involving others.
3.1.3 Cultural barriers toward caregiver support and counseling	3.2.2 Culturally sensitive language adjustments	Reframed counseling in familiar terms; emphasized stress management and self‐care to reduce stigma.
3.1.4 Concerns about privacy and study participation	3.2.4 Adapting delivery methods	Provided home‐based visits and alternatives to group‐based formats to address privacy and disclosure concerns.
3.1.5 Blood test and health‐related concerns	3.2.4 Adapting delivery methods	Used dry blood spot cards and conducted at‐home collection with explanations in native language to ease participant anxiety.
Cross‐cutting across all challenges	3.2.1 Community partnerships	Engaged culturally aligned community organizations (e.g., CaringKind, Korean Community Services of Metropolitan New York) to build trust and enable tailored outreach.

#### 3.2.1 The critical role of community partnerships and engagement in recruitment

Building and strengthening community partnerships was a cornerstone of our recruitment strategy for both Chinese and Korean American caregivers. These partnerships, combined with participation in community events and workshops, played an essential role in fostering trust, promoting our study, and engaging potential participants. An important aspect of our recruitment success involved securing additional funding for community organizations. Recognizing the critical role these organizations played in recruitment, we allocated funds, not originally proposed in the budget, to support staff efforts in helping to recruit participants. This funding ensured that community partners had the resources necessary to dedicate time and effort to referral, recruitment, and outreach activities, further enhancing our ability to connect with potential participants.

For Chinese American recruitment, we leveraged existing relationships with organizations like CaringKind, which allowed us to promote the study through Chinese newspapers, community events, and referrals from local agencies. CaringKind also facilitated new connections with other Chinese community agencies, expanding our recruitment network even further. Additionally, we conducted educational workshops focusing on practical caregiving skills. These workshops were held in the participants' preferred language, making them more accessible, particularly for older caregivers. The familiar setting of these events further helped us build rapport and engage the community.

For Korean American recruitment, we initially lacked strong connections within the community. However, we nurtured new partnerships, with KCS becoming our primary collaborator. KCS played a critical role in identifying and referring participants, promoting our study during community events and classes. Despite outreach to other organizations like the Asian Women's Christian Association (AWCA), many directed us back to KCS, recognizing it as the largest organization with the resources needed for effective recruitment. Our participation in KCS‐sponsored brain health and mental health awareness events helped us build trust within the community, allowing us to reach caregivers who might not have been accessible through formal channels.

In both Chinese and Korean American communities, community partnerships and engagement were integral to our recruitment efforts. These collaborations not only expanded our network but also allowed us to engage caregivers in a culturally appropriate and accessible manner, underscoring the utmost significance of community involvement in achieving recruitment success.

#### 3.2.2 Culturally sensitive language adjustments to increase engagement

To reduce resistance, we replaced the term “counseling” with “family meetings,” which better aligns with cultural norms and makes the concept more approachable. We also toned down the formal “research” language and personalized the message, using layman's terms and framing the outreach in a community‐friendly way. Many Chinese American caregivers associated counseling with stigma or unfamiliarity but positioning the sessions as family gatherings helped overcome these barriers. Additionally, presenting these meetings as opportunities for children to practice filial piety, engage with their parents’ health, and plan for future care significantly reduced initial rejection rates. When reframed in this way, participants were more open to the idea of participating in these sessions. Additionally, during initial recruitment calls, we found it crucial to take the time to listen to and validate caregivers’ thoughts and concerns to build trust. Instead of emphasizing potential depression, we explained that the sessions and social media groups were designed to help caregivers manage stress and focus on self‐care, which is more culturally acceptable. We also highlighted access to community resources that caregivers might not be aware of or may be hesitant to use, further encouraging their engagement in the program. For Korean American caregivers, we replaced “counseling” with culturally appropriate phrases like “talk about caregiving burden” or “learn about caregiving strategies.” These terms resonated more with participants, as they emphasized the practical aspects of caregiving rather than the stigma associated with counseling. This adjustment significantly reduced participant hesitancy and made the sessions feel more accessible and less emotionally demanding.

#### 3.2.3 Reframing participation as culturally meaningful

For both Chinese and Korean American caregivers, we emphasized culturally meaningful reasons for participating in the study, though the specific values highlighted differed between the groups.

Chinese American caregivers were more open to participation when we framed family meetings as opportunities for children to practice filial piety—a key cultural value. This reframing helped caregivers view participation as an important family responsibility rather than a personal burden.

For Korean American caregivers, we emphasized both personal and social benefits. On the personal side, participants valued the at‐home health check‐ups, the chance to share their caregiving stories, and the financial incentives. On the social side, many caregivers were motivated by the idea that their participation could influence future policies on dementia caregiving, which could benefit the broader Korean immigrant community. This sense of contributing to society and the next generation of Korean Americans encouraged participation.

#### 3.2.4 Adapting delivery methods for accessibility and participant engagement

Adapting our study procedures to accommodate participants’ preferences and concerns was essential for improving engagement among both Chinese and Korean American caregivers. These adjustments not only helped mitigate initial challenges but also enhanced participation, offering additional benefits to the caregivers.

For example, blood drawing was initially a concern for many participants. To address this, we adapted by offering at‐home visits for data collection, which significantly alleviated concerns. This change transformed a potential barrier into a motivating factor for some participants. In consultation with other researchers, we further modified our procedure by using dry blood spot (DBS) cards, which require only 9 to 12 drops of blood, rather than collecting whole blood as originally planned. During home visits, social workers collected the samples and handled shipping on behalf of the participants. As a result, the number of participants agreeing to the blood test increased in both the Chinese and Korean American groups. Some participants, particularly those without health insurance, viewed the blood test as an opportunity to catch up on health check‐ups they had missed. They appreciated the health information provided by the study, including follow‐up calls to discuss their results. Participants also shared that, in previous experiences with blood work, they often received reports in English with little explanation unless something concerning was found. In our study, student workers and social workers translated the results and took time to explain the findings, which participants found particularly valuable.

To ensure accessibility for older participants, we made specific adaptations. For Chinese American caregivers, we offered the option to use WeChat video calls instead of Zoom, as WeChat was a more familiar platform for many. This adjustment reduced the stress of navigating unfamiliar technology and made it easier for caregivers to participate in family meetings. For Korean American caregivers, we found that home visits were more effective and acceptable. Many caregivers found in‐person visits more convenient and were more likely to engage when the study activities were conducted in their homes. These adaptations, both in health‐related procedures and technology use, played a crucial role in making the study more accessible, reducing participant stress, and encouraging engagement.

### 3.3 Recruitment outcomes

From February 2023 to the end of June 2025, we used multiple strategies to recruit participants, as outlined in Table 1. Our primary strategy was collaborating with community organizations. Among the 288 individuals who were enrolled, 204 heard about our study through community organizations and participated (70.83%). Specifically, we partnered with organizations serving Chinese and Korean American immigrant populations. Through these resources, we have successfully enrolled 197 participants, including 75 Chinese and 122 Korean Americans. We also connected with local hospitals and clinics and identified nine eligible individuals during screening, resulting in the enrollment of an additional seven Chinese American participants. Other methods contributed smaller but meaningful numbers. Word‐of‐mouth referrals, including informal conversations with participants, study personnel, and community members, helped us enroll 55 participants (19.10%). Community outreach efforts—such as educational workshops, volunteer activities, and participation in community events—resulted in 21 enrollments (7.29%). Social media advertisements, including those via newspapers and online platforms, led to recruiting six eligible individuals and the enrollment of five (2.58%). Last, other strategies, such as distributing flyers through local public venues (e.g., churches, libraries), facilitated the enrollment of two additional participants (0.69%).

**FIGURE 1:**
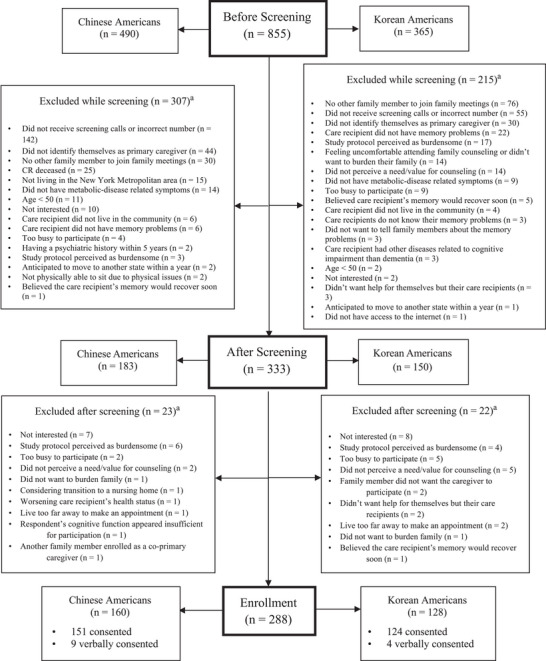
Recruitment status and the reasons for non‐participation. Note: Participants could address multiple reasons for exclusion. CR, care recipient.

**FIGURE 2:**
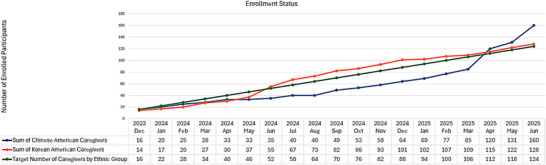
Enrollment status between December 2023 and March 2025.

During the recruitment phase, we conducted initial screening calls with 490 Chinese American and 365 Korean American individuals. Subsequent screenings led to the exclusion of 307 Chinese Americans and 215 Korean Americans for various reasons, as shown in Figure 1. The three most common reasons for exclusion were: non‐responsiveness to screening calls (142 Chinese and 55 Korean Americans), not identifying as the primary caregiver (44 Chinese and 30 Korean Americans), and lack of another family member able to participate in family meetings (30 Chinese and 76 Korean Americans). Among Chinese Americans, additional reasons for ineligibility included the care recipient's passing (*n* = 25); not living in New York Metropolitan area (*n* = 15); or absence of cardiometabolic conditions such as diabetes, hypertension, hyperlipidemia, or overweight/obesity (*n* = 14). In contrast, Korean Americans more frequently cited reasons such as care recipients not exhibiting memory symptoms (*n* = 22), the perceived burden of the study protocol (*n* = 14), discomfort with participating in family counseling sessions (*n* = 17), and discomfort with participating in family counselling sessions or burdening their family (n = 14). Additional reasons for exclusion are detailed in Figure 1. After the screening process, 333 individuals were deemed eligible: 183 Chinese and 150 Korean Americans. By June 2025, 288 participants have been enrolled, including 160 Chinese and 128 Korean Americans. Figure 2 illustrates the progression of enrollment status between 2023 and 2025.

We compared individuals who were eligible but did not enroll (*N* = 45) with those who were eligible and did participate (*N* = 288) to explore whether certain characteristics were associated with participation (see Table 2). According to screening questionnaires, there were no statistically significant differences between the two groups across any measured variables. The mean age of caregivers was similar (65.97 years for non‐participants vs. 68.63 years for participants), with a high percentage of female caregivers in both groups (79.55% vs. 83.33%). Spousal caregivers were more likely to participate in the study than not (42.22% vs. 55.90%). Additionally, the presence of cardiometabolic risk factors (diabetes, high cholesterol, hypertension, and obesity) showed mixed patterns. There were no statistically significant differences in the prevalence of diabetes or high cholesterol. However, eligible but non‐participants were significantly less likely to report hypertension and overweight (see Table 2).

Table 3 presents the demographic and health characteristics of the 288 enrolled caregivers. The average age was 69.22 years (standard deviation [SD] = 8.91) for Chinese American caregivers and 67.89 years (SD = 9.96) for Korean American caregivers. The majority of caregivers in both groups were female (83.12% of Chinese American caregivers and 83.59% of Korean American caregivers) and were providing care to a spouse or partner (59.38% vs. 51.56%). In terms of cardiometabolic risk factors, the most reported condition in both groups was high cholesterol, while the least reported was diabetes among Chinese participants and overweight or obesity among Korean participants. A significantly higher percentage of Chinese American caregivers reported diabetes and overweight or obesity compared to Korean American caregivers (44.38% vs. 27.34%, *t* = 8.8683, *p* = 0.003; 64.38% vs. 25.00%, *t* = 44.2729, *p* < 0.001). Additionally, a significantly higher percentage of Chinese American caregivers reported having multiple health conditions compared to their Korean American counterparts.

The purpose of the comparisons in Tables 2 and 3 is to provide context about the study sample and explore potential differences between those who enrolled and those who did not. These comparisons are intended to be descriptive rather than inferential, offering readers a clearer understanding of participation patterns. Specifically, this comparison helps evaluate the potential for selection bias, that is, whether individuals who chose to participate differed systematically from those who were eligible but did not enroll.

## 4 DISCUSSION

This study demonstrates the complexity and importance of adapting recruitment and strategies for culturally diverse populations, specifically Chinese and Korean American dementia caregivers. Despite our initial focus on cultural sensitivity and inclusivity, we encountered challenges related to cultural barriers and logistical concerns during recruitment. However, through strong community partnerships, culturally adapted communication, and iterative refinements based on timely and continuous communication between the principal investigators and the research staff, we successfully engaged many participants. Our findings underscore the critical need for tailoring recruitment approaches to reflect the cultural context of target communities.

As of this writing, we have successfully enrolled 288 participants (160 Chinese and 128 Korean Americans), a notable increase from the 124 participants reported 5 months ago. This growth reflects the strength of our personalized and sustained engagement strategies. Our recruitment outcomes align with and extend the literature on engaging Asian American populations in dementia research. The University of California San Francisco Alzheimer's Disease Center successfully recruited 125 Chinese American participants over several years through community clinics and events, largely driven by word of mouth and clinical referrals. ^35^ Unlike large‐scale efforts,^36^, ^37^ our approach was highly targeted and focused specifically on Chinese and Korean American primary dementia caregivers with elevated cardiometabolic risk. These communities are particularly difficult to reach due to cultural stigma, time constraints, competing family obligations, and the extended 12 month commitment required by our intervention study. Our findings are further supported by a series of recent studies by Liu et al., who examined the experiences and service needs of Chinese American dementia caregivers.^20^, ^21^, ^22^ Culturally tailored outreach, peer‐based trust, and community partnerships were central to their recruitment efforts. These strategies resonate with our own experiences, particularly the importance of bilingual staff, relationship building, and direct referrals. Like Liu's teams, we found that passive strategies such as ethnic media advertisements were far less effective for reaching this population, underscoring the need for active, personalized approaches—especially when engaging caregivers managing multiple demands and participating in long‐term interventions.

Our pre‐intervention focus groups with Chinese and Korean dementia caregivers provided invaluable insights that shaped the development of our culturally tailored approach.^30^ These focus groups revealed that cultural and family expectations strongly influenced caregivers' roles, their emotional well‐being, and the specific barriers they encountered in accessing health care for themselves and their relatives with dementia. Caregivers often faced stigma, deeply rooted in cultural norms, which impacted their perceptions and use of support systems. Furthermore, we found that many caregivers were open to the potential benefits of telehealth and social media platforms in managing dementia care. These insights directly informed the design of our recruitment and intervention protocols, ensuring a person‐centered, culturally appropriate approach.

Despite these initial efforts, we encountered challenges during the implementation phase. Messaging and terminology presented difficulties, underscoring the iterative nature of tailoring recruitment strategies. Our approach aligns with key principles from culturally tailored interventions, which emphasize adapting interventions to fit cultural and contextual needs.^38^, ^39^, ^40^ Previous research has highlighted the importance of community partnerships in recruiting Asian American populations, bilingual staff, and cultural competence.^13^, ^14^

Specifically, we secured additional funding to support community partners. Recognizing the pivotal role these organizations played, we allocated resources to assist their staff efforts, which allowed them to focus on referral, recruitment, and outreach activities. Notably, many of these community organizations operate with limited resources, making this support essential. By partnering with organizations like CaringKind and KCS, we established reciprocal relationships that extended beyond recruitment into active community engagement. These reciprocal relationships involved mutual support, by which we provided financial and logistical assistance, and in return, the organizations offered their cultural expertise and trusted community connections, ensuring that both parties benefited. Our support also helped these organizations make a greater impact in their communities by enhancing their capacity to provide services and reach more individuals. This collaboration facilitated study promotion and increased participant trust.

We built a bilingual and bicultural research team to foster culturally sensitive communication, and our findings expand on previous research by addressing the unique challenges faced by Chinese and Korean American dementia caregivers. Cultural competence in our study extended beyond language proficiency to a nuanced understanding of caregiving dynamics within each community. We aligned our language with cultural norms, reducing stigma around counseling services and facilitating participation. The linguistic adaptation was significant in overcoming cultural resistance to mental health services. Similarly, understanding the deep‐rooted attitudes toward caregiving responsibilities in these communities allowed us to position our interventions in ways that resonated with participants’ values and beliefs, particularly around family roles, filial expectations, and social support.

Our findings also add to the understanding of acculturation differences within Chinese American caregiving populations. A major theme throughout our recruitment efforts was the reluctance among caregivers, particularly Korean Americans, to involve family members in counseling sessions. Similar to findings from Kwon et al.^13^ and Lee,^41^ many participants were hesitant to involve their children, fearing it would burden them or disrupt family dynamics. This may also reflect cognitive acculturation, in which older immigrants cultivated individualism and valued independence.^42^ In response, we allowed participants more time (2–4 weeks after enrolling) to decide which family members to involve, ensuring they were comfortable with their decisions. As trust grew over time, caregivers became more open to participating in family‐based interventions, suggesting that future studies could benefit from a longer lead‐in period to build rapport before introducing family‐based components.

We implemented a culturally sensitive recruitment strategy that combined planned adaptations with responsive adjustments based on participant feedback. Drawing on insights from pre‐intervention focus groups, we refined study messaging to reduce stigma and enhance relevance. Some modifications emerged during implementation; for example, several Korean American caregivers expressed reluctance to involve their children in family sessions, citing a desire to avoid burdening them, a theme reflected in prior research on intergenerational caregiving dynamics.^20^, ^21^, ^22^ In these cases, we adjusted the timeline to build trust and allow participants more time to consider family involvement. These adjustments align with the Framework for Reporting Adaptations and Modifications–Enhanced (FRAME) framework,^43^ which highlights the distinction between planned and reactive modifications. Overall, our approach underscores the importance of cultural competence and responsiveness in recruitment for community‐based dementia research.

One distinctive feature of our study was the inclusion of biomarker data collection alongside a psychosocial intervention, which expanded the scope of traditional caregiver‐focused research. While the addition of biological sampling, specifically DBS cards for cardiometabolic and inflammatory markers presented initial concerns for some potential participants, these concerns were largely alleviated through careful, culturally sensitive framing. Several individuals initially expressed hesitation about giving a blood sample, reflecting broader trends in the literature that highlight wariness among older adults in some Asian American subgroups regarding unfamiliar biomedical procedures.^44^ To address this, we emphasized the voluntary nature of biomarker participation and clearly communicated that caregivers could still fully participate in the study without providing a biological sample. We also minimized barriers by offering convenient, minimally invasive finger‐prick testing and optional at‐home collection visits. These adaptations helped ease logistical concerns and built trust. Ultimately, many participants welcomed the opportunity to receive health information such as cholesterol or glucose levels. In this way, the biomarker component served as both a recruitment challenge and an engagement opportunity, underscoring the importance of clear communication and participant‐centered design when integrating biomedical elements into community‐based research.

Our findings align with previous research involving other Asian American communities, such as Vietnamese and South Asian American populations, which similarly underscore the importance of trust building, cultural nuance, and community partnerships in recruitment for aging and dementia research.^36^, ^45^, ^46^ For example, recruitment among Vietnamese caregivers often relied on long‐standing relationships with key informants and community‐based organizations, requiring years of engagement and bilingual staffing to overcome initial distrust.^46^ Similarly, in studies with South Asian older adults, collectivist values influenced decision making, with many older adults seeking permission from adult children before participating, and confidentiality concerns remained significant. Across these studies, passive outreach methods (e.g., flyers or social media) were less effective than active, relationship‐based recruitment through trusted community channels. Our study extends these findings by emphasizing the use of culturally sensitive language, reframing research participation as culturally meaningful, and leveraging bilingual social workers for sustained engagement. These strategies may enhance recruitment feasibility and ethical alignment across other understudied Asian American groups in dementia research.

Our study contributes key insights to the literature on recruitment strategies for Asian American dementia caregivers, emphasizing the need for continuous adaptation of strategies to meet cultural needs. Cultural competence is not a static process but requires ongoing refinement to ensure that language, technology, and communication methods remain relevant and effective. Moreover, our findings highlight the role of acculturation in shaping openness to research participation and mental health services, which future studies must consider when designing interventions for culturally diverse populations. By adopting an iterative process of adapting recruitment and intervention strategies based on real‐time feedback, we provide a model for ensuring that interventions remain culturally sensitive and accessible. As cultural adaptation is an ongoing process, it is essential to document and report any modifications to evidence‐based interventions to maintain transparency and fidelity.^43^ While this study provides valuable insights into recruitment strategies for Chinese and Korean American dementia caregivers, we acknowledge that the findings do not fully capture the cultural diversity within these communities. The perspectives and responses of participants in our study reflect the specific geographic, linguistic, and community contexts in which recruitment occurred and should not be assumed to represent all Chinese or Korean American populations.

## 5 CONCLUSIONS

This study demonstrates our ongoing efforts and success in recruiting and retaining Chinese and Korean American dementia caregivers, while also highlighting the complexities involved in culturally tailored interventions. Through strong community partnerships, culturally adapted communication, and iterative refinements, we have successfully engaged many participants and continue to actively recruit. By sharing our interim results and experiences, we offer valuable insights into the importance of cultural competence, flexibility, and responsiveness in research with diverse populations. These insights provide a practical model for future research targeting underrepresented dementia caregiver populations with cardiometabolic risks.

## CONFLICT OF INTEREST STATEMENT

All authors declare that they have no conflicts of interest to disclose. Author disclosures are available in the supporting information.

## CONSENT STATEMENT

All human subjects included in this study provided informed consent

## Supporting information



Supporting Information
